# Whole genome characterization and diagnostics of prunus necrotic ringspot virus (PNRSV) infecting apricot in India

**DOI:** 10.1038/s41598-023-31172-z

**Published:** 2023-03-16

**Authors:** Md Salik Noorani, Mirza Sarwar Baig, Jawaid Ahmad Khan, Alam Pravej

**Affiliations:** 1 Department of Botany, School of Chemical and Life Sciences, Jamia Hamdard (A Deemed-to-Be University), New Delhi, India; 2 Department of Molecular Medicine, School of Interdisciplinary Sciences, Jamia Hamdard (A Deemed-to-Be University), New Delhi, India; 3 Plant Virus Laboratory, Department of Biosciences, Jamia Millia Islamia (A Central University), New Delhi, India; 4 Biology Department, College of Science and Humanities, Prince Sattam Bin Abdulaziz University (PSAU), 11942 Alkharj, Kingdom of Saudi Arabia

**Keywords:** Microbiology, Virology, Viral evolution

## Abstract

Prunus necrotic ringspot virus (PNRSV) is a pathogen that infects *Prunus* species worldwide, causing major economic losses. Using one and two-step RT-PCR and multiplex RT-PCR, the whole genome of the PNRSV-infecting apricot was obtained and described in this study. Computational approaches were used to investigate the participation of several regulatory motifs and domains of the Replicase1, Replicase2, MP, and CP. A single degenerated reverse and three forward oligo primers were used to amplify PNRSV’s tripartite genome. The size of RNA1 was 3.332 kb, RNA2 was 2.591 kb, and RNA3 was 1.952 kb, according to the sequencing analysis. The Sequence Demarcation Tool analysis determined a percentage pair-wise identity ranging between 91 and 99% for RNA1 and 2, and 87–98% for RNA3. Interestingly, the phylogenetic analysis revealed that closely related RNA1, RNA2, and RNA3 sequences of PNRSV strains from various geographical regions of the world are classified into distinct clades or groups. This is the first report on the characterization of the whole genome of PNRSV from India, which provides the cornerstone for further studies on the molecular evolution of this virus. This study will assist in molecular diagnostics and management of the diseases caused by PNRSV.

## Introduction

Apricot (*Prunus armeniaca* L.) is a popular fruit of the family Rosaceae that is widely cultivated commercially in many countries, including India and around the world especially in temperate climates. It is consumed fresh, dried, or processed into various food products. The apricot industry is a key sector of agriculture in many countries, creating employment and contributing to local and national economies. However, like any other crop, apricot production can be affected by various factors, such as changes in weather patterns, diseases and pests, which can threaten the productivity and profitability of the industry.

Viral diseases are one of the most significant obstacles to apricot production. It is a serious threat to apricot as well as other stone fruit industries that can cause significant yield losses and impact the overall fruit quality^1^. Plant virologists have developed several cost-effective and faster nucleic acid-based diagnostic assays to detect many viruses simultaneously from infected plants over the past few decades^2^. Multiplex RT-PCR (mRT-PCR) is one of them which is used to amplify more than one target at the same time in a single PCR reaction by using multiple primer pairs that are specific to each target^3^. There are two methods for mRT-PCR: one-step (cDNA synthesis and PCR are combined in a single tube) and two-step (cDNA synthesis and PCR are performed in a separate tube. Both approaches have proven useful for identifying many viruses, viroid, and phytoplasma species from the same or distinct genera simultaneously^2^.

Prunus necrotic ringspot virus (PNRSV: Genus *Ilarvirus*, Family *Bromoviridae*) causes ringspot disease. *Prunus* species, both cultivated and wild, as well as other plants such as roses (*Rosa* spp.), apples (*Malus domestica*) and hops (*Humulus lupulus*) are all vulnerable to single or multiple strains of PNRSV^1,4^. PNRSV has a segmented, tripartite, positive-sense, single-stranded (ss) RNA genome consisting of RNA1 (3.3 kb), RNA2 (2.5 kb), and RNA3 (1.9 kb). The 3′ end of each genomic segment is extremely conserved, while the 5′ end is less so. These three genomic RNAs serve as messenger (m) RNAs. Replication protein (1a) and RNA-directed RNA polymerase (2a) encoded by mRNA1 and mRNA2, respectively, are involved in replication and internal transcription of subgenomic RNA3b. Movement and capsid proteins are produced by subgenomic RNA3a and RNA3b, respectively^5^.

PNRSV is widely distributed worldwide due to its ease of transmission via plant propagation methods, contaminated pruning tools, seeds and pollen^6^. Infected plants may be asymptomatic or exhibit symptoms including necrotic shock, chlorotic and necrotic leaf spots, rugose mosaics, yellow line patterns, cherry bud death, and discolored rings or patches on apricot fruits. Infected trees also show poor bud growth, fruit vigour, and quality, as well as being more susceptible to cold^7^. PNRSV produces significant economic losses and has an impact on overall fruit production and quality in susceptible hosts^1,8^. During a recent survey, PNRSV was reported to be prevalent in up to 98% of the leaf samples taken from apricot growing at Srinagar and Shopian districts of Jammu and Kashmir, India^9^.

In an era of climate change and global trade, finding effective diagnostic techniques for plant virus identification and management is crucial. A vast number of viruses are spreading worldwide with people and commodities, causing serious agricultural problems^10,11^. Thus, early identification of plant viruses is essential in plant health monitoring to manage disease infections at various stages, reduce disease spread, and prevent new infections^12^. Further, whole-genome sequencing (WGS) of viruses is becoming more significant in today's world for basic and advanced research. It makes a substantial contribution to the development of novel vaccines, the advancement of molecular epidemiology, and pathogen control approaches. The WGS foundation is critical for understanding the genomic diversity, evolutionary history, and biological functions of viruses^13^.

In this work, a one-step and two-step mRT-PCR has been developed to amplify and characterize the whole genome of PNRSV infecting apricot from India and compared these RNAs (RNA1, RNA2 and RNA3) sequences with other available PNRSV genomic sequences from different geographical locations of the world. In addition to molecular characterization, in silico analysis such as pair-wise sequence comparison, percent nucleotide identity, phylogenetic relationship, and recombination analysis are discussed for identifying genetic variations in PNRSV isolates.

## Materials and methods

### Sampling area

Fifty two samples collected by Noorani and Khan^14^ and Noorani et al.^9^ from several apricot-growing orchards in Srinagar near the Jhelum river (Latitude: 34° 5′ 1.1616′′ N and Longitude: 74° 47′ 50.5356′′ E) districts of Jammu and Kashmir, in the North of India, were used for the development of multiplex RT-PCR and whole genome characterization of PNRSV.

### Total RNA isolation

Following maceration in liquid nitrogen, total RNA was isolated from 100 mg of leaf tissues from infected and healthy plants. The standard CTAB approach was used for RNA isolation in the procedure as earlier described by Noorani et al.^15^. The quantity and purity of RNA was measured with nanodrop (Eppendorf-Biophotometer, Germany), and non-degenerating agarose gel electrophoresis was used to determine the integrity.

### Primer design and synthesis

Separate forward primers for RNA1, RNA2, and RNA3 and a single reverse degenerate primer for all three RNAs were designed and used to standardize RT-PCR and mRT-PCR to amplify the whole/partial length of the tripartite genome of PNRSV. The sequences were retrieved from the NCBI (http://www.ncbi.nlm.nih.gov/) for multiple sequence alignment and primer designing. The primers’ physical properties and internal structures, such as hairpins, self and heterodimers, were examined using the OligoCalc software (http://www.basic.northwestern.edu/biotools/oligocalc.html)^16^. These primers were synthesized by GCC Biotech (Kolkata, India). The primers used for cDNA synthesis and PCR are listed in Table 1.

**Table 1:** Primers used for the amplification of the whole genome of PNRSV by one and two-step RT-PCR and mRT-PCR in this study.

Primer Name	Oligo Sequence (from 5′ to 3′)	Target	Product size (bp)
PNRNA1F	ACGGTTTTTAGTTCGTGGTTGAAC	RNA1	3332
PNRNA123R	GCTTCCCTATCCGGGCATCYACRACTTC		
PNRNA2F	GTTTTTGTACTCGTGGTTGAGTTAC	RNA2	2591
PNRNA123R	GCTTCCCTATCCGGGCATCYACRACTTC		
PNRNA3F	GTTTTAACCTAATTGAAATCGTAACATCC	RNA3	1952
PNRNA123R	GCTTCCCTATCCGGGCATCYACRACTTC		

### cDNA synthesis for two-step RT-PCR and mRT-PCR

The cDNA was synthesized using a ProtoScript AMV LongAmpTaqRT-PCR Kit (New England Biolabs, USA) following the manufacturer’s instructions for two-step RT-PCR and mRT-PCR. Briefly, 10 µl of reaction mixture containing PNRNA123-R reverse primer and 400 ng total plants RNA were incubated at 42 °C for 55 min, followed by 5 min at 80 °C and were kept at − 20 °C for later use.

### One and two-step RT-PCR

RT-PCR was performed for each of PNRSV’s three RNAs using corresponding primer pairs to evaluate primer efficacy, specificity and to establish optimal PCR conditions.

In one-step RT-PCR, a total of 10 µl of reaction volume containing 1 µl RNA (400 ng), 5 µl OneTaq One-Step Reaction Mix (2×), 0.4 µl OneTaq One-Step Enzyme Mix (25×) of OneTaq One-Step RT-PCR Kit (New England Biolabs, USA), 0.25 µl (from 10 pM) each forward and reverse primer was prepared. The PCR reaction was set up in separate thin-walled 200 μl tubes for each RNA fragment in a thermocycler 8800 (Agilent Technologies, USA), The cycling conditions were reverse transcription at 48 °C for 30 min, initial denaturation at 94 °C for 1 min, 33 cycles of denaturation at 94 °C for 30 s, annealing at 51 °C for 30 s, and extension at 68 °C for 3.20 min. The final step of the extension was 68 °C for 10 min.

For two-step RT-PCR, 10 µl reaction containing 1 µl cDNA, 5 µl 2× master mix of ProtoScript AMV LongAmpTaq RT-PCR Kit (New England Biolabs, USA), 0.25 µl (from 10 pM) each forward and reverse primer and 4.5 µl of nuclease-free water, was set up for each RNA fragment in a thermocycler 8800 (Agilent Technologies, USA). Gradient PCR was used to optimize conditions, with one cycle at 94 °C for 1 min, 35 cycles at 95 °C of final denaturation for 30 s, annealing temperatures of 50–55 °C for 30 s, and extension at 68 °C for 2–3.20 min followed by one cycle of final extension at 68 °C for 10 min.

### One and two-step mRT-PCR

All four primers (PNRNA1-F, PNRNA2-F, PNRNA3-F, and PNRNA123-R) were utilized in a single tube to amplify all three RNA fragments of PNRSV concurrently in one and two-step mRT-PCR. In one-step mRT-PCR, a total of 10 µl of reaction was set up in a single PCR tube using total plant RNA (400 ng), three forward (0.25 uM each), and one reverse (0.75 uM) primer together with all of the components of the OneTaq One-Step RT-PCR Kit (New England Biolabs, USA) in a thermocycler 8800 (Agilent Technologies, USA). Cycling conditions were identical to those employed in one-step RT-PCR.

In two-step mRT-PCR, 10 µl reaction containing 1 µl cDNA, 5 µl master mix (2×) of ProtoScript AMV LongAmpTaq RT-PCR Kit (New England Biolabs, USA), 0.25 µl (from 10 pM) each three forward, 0.75 µl (from 10 pM) of reverse primer and 2.5 µl of nuclease-free water, was set up in a single PCR tube in a thermocycler 8800 (Agilent Technologies, USA). The PCR amplification was performed with a 1 min denaturation step at 95 °C followed by 35 cycles of 95 °C for 30 s, 51 °C for 30 s, 68 °C for 3.20 min and a final extension at 68 °C for 10 min.

### Sensitivity of one and two-step RT-PCR and mRT-PCR

RNA1, RNA2 and RNA3 concentrations were adjusted to 400 ng/reaction (10^0^) for the sensitivity of RT-PCR and mRT-PCR in one and two-step. Furthermore, the detection limits of various RNA fragments were determined using ten-fold serial dilutions (10^0^–10^–5^) of RNA1, 2 and 3.

### Nucleotide sequencing

PCR products were electrophoresed in a one percent agarose gel containing ethidium bromide (0.5 g/ml) and photographed under gel documentation system (Bio-Rad Laboratories, USA). Following electrophoresis, the gel-excised products were purified according to the manufacturer's recommendations using the QIAquick Gel Extraction Kit (Qiagen, Germany). Sanger sequencing methods^17^ directly sequence the purified PCR amplicons. The BLASTn tool^18,19^ corroborated the sequences obtained by comparing them to a published database accessible in NCBI-GenBank.

### Computational analysis of whole-genome of PNRSV

The other highly conserved full-length PNRSV RNA1, RNA2, and RNA3 nucleotide sequences were identified by the BLAST 2.8.1 program available at NCBI^18,19^. RNA1, RNA2, and RNA3 sequences of Indian isolate under study were used as a query in the nucleotide-BLAST, and the resulting seventeen, eighteen and twenty-nine other highly conserved full-length PNRSV RNA1, RNA2 and RNA3 sequences respectively from different isolates were retrieved from NCBI.

Motifscan^20^ and MotifFinder^21^ programs were applied for the identification of potential regulatory motifs in the Replicase1, Replicase2, movement protein, and coat protein-encoding RNA sequences of PNRSV^22^. Different motif databases like HAMAP profiles^23^, PROSITE patterns, PROSITE profiles^22,24^, Pfam HMMs (local), and Pfam HMMs (global) were searched to predict motifs. Meanwhile, HMMER^25^, Pfam^26^, and NCBI-CDD^27^ online tools were used to identify the significant conserved domains^27^. NCBI-CDD with default setting was searched for the prediction of conserved domains. The threshold E-value was selected at 0.01 in all these three online domain prediction tools. The molecular weight, isoelectric point, stability index, and hydrophobicity of the amino acid sequences of Replicase1, Replicase2, movement protein and coat protein-encoding RNA sequences of PNRSV were predicted using ExPASy protparam server.

### Sequence demarcation tool (SDT)

SDT is a computer program that provides a robust virus classification tool based on pairwise sequence alignment and genetic identity calculations to classify any set of nucleotide or amino acid sequences^28^. SDT supports the classification of sequences according to ICTV recommended species demarcation criteria and produces identity plots and colour-coded distance matrices. ClustalW^29^ aligned PNRSV genomic RNA1, RNA2, and RNA3 sequences and were further analyzed in the sequence demarcation tool version 1.2 (SDTv1.2) to determine the percent nucleotide identity.

### Phylogenetic analysis

Phylogenetic trees based on the Neighbor-Joining method were constructed using MEGA11^30^. MEGA11 was also used to build pairwise, multiple sequence alignments, and visualize the tree. In total, 17 (RNA1), 18 (RNA2), and 28 (RNA3) complete putative sequences were shortlisted for this study from the NCBI database along with an understudy Indian isolate of PNRSV. The prune dwarf virus (RNA1: Acc. No. NC008039, RNA2: Acc. No. NC008037 and RNA3: Acc. No. NC008038) was used as an out-group in phylogeny reconstruction and sequence comparisons. To infer the robustness of evolutionary relationships, the consensus trees were built using 1000 bootstrap replicates.

### Recombination analysis

To assess the genetic variability in the genomic RNA sequences of the PNRSV of apricot (under study), with other PNRSV sequences available at NCBI were analyzed by Recombination Detection Program version 4.97 (RDP Beta 4.97)^28^. The RDP4 Beta 4.97 finds the putative recombination hotspots and parental sequences in these PNRSV sequences. Nine recombination detection and analysis methods viz., RDP, GENECONV, Bootscan, MaxChi, Chimaera, SisScan, PhylPro, VisRD, and LARD were implemented by default parameters of the highest acceptable probability value (*P* value) of 0.05.

### Statement

Experimental research and field studies on cultivated plants, including the collection of plant material, are complied with relevant institutional, national, and international guidelines and legislation.

## Results

### Amplification of the whole genome of PNRSV by one-step and two-step RT and mRT-PCR

**Figure 1:**
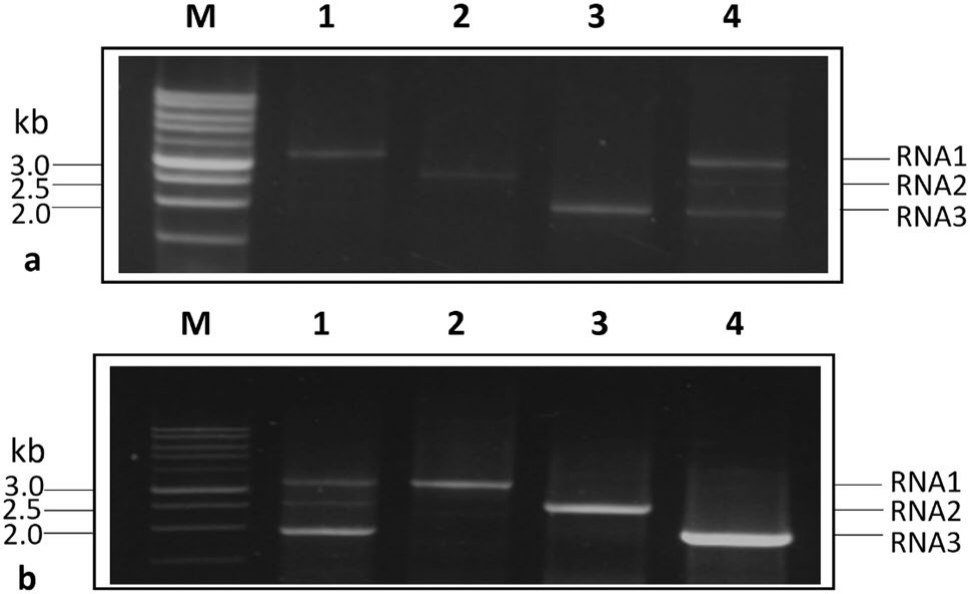
Amplification of whole genome of PNRSV by (**a**) one-step RT-PCR: Lane 1, 2 and 3 showing amplification of RNA 1, RNA 2 and RNA 3 respectively. Lane 4 is showing simultaneous amplification of RNA1, 2 and 3 by one-step mRT-PCR. (**b**) two-step RT-PCR: Lane 2, 3 and 4 showing amplification of RNA 1, RNA 2 and RNA 3 respectively. While Lane 1 shows simultaneous amplification of RNA 1, 2 and 3 by two-step mRT-PCR. Lane M showing 1 kb DNA ladder (RTU, GeneDireX, Taiwan).

The whole genome of PNRSV, i.e., RNA1, RNA2, and RNA3, was successfully amplified employing one-step RT-PCR along with mRT-PCR (Fig. 1a, Supplementary Fig. S1a) and two-step RT-PCR along with mRT-PCR (Fig. 1b, Supplementary Fig. S1b).

### Sensitivity of one-step RT-PCR and mRT-PCR

**Figure 2:**
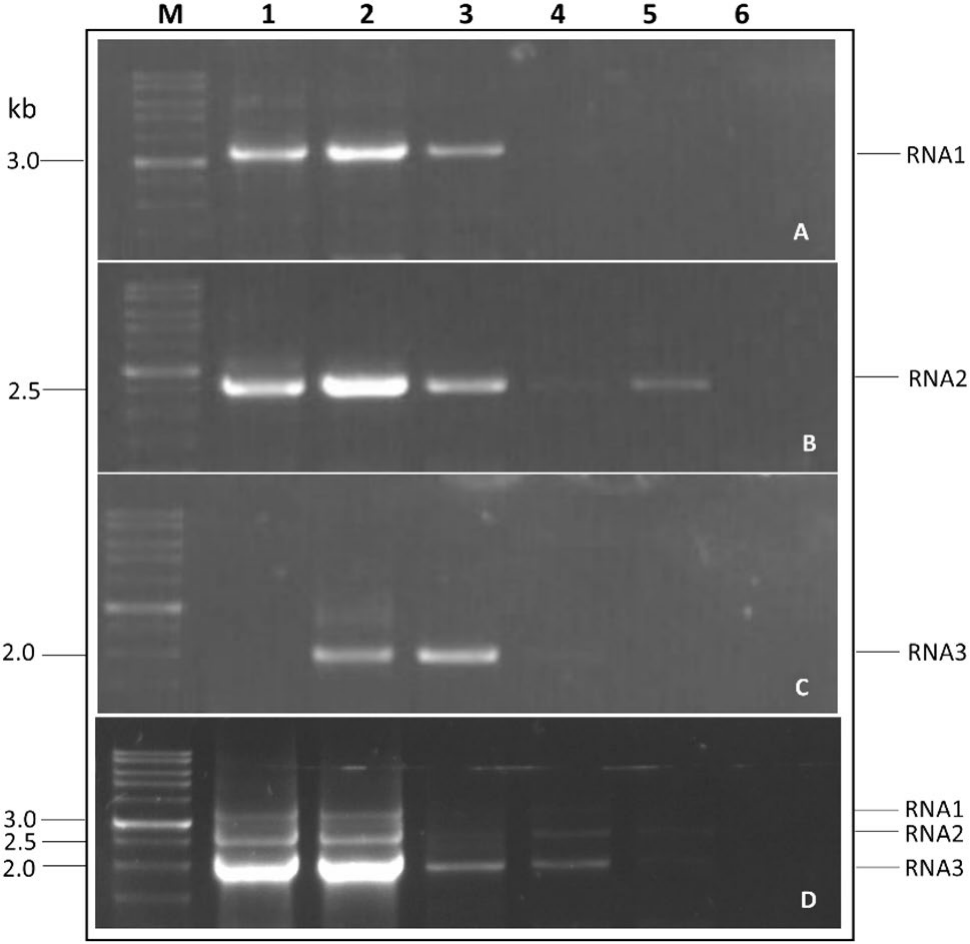
Sensitivity of one-step RT and mRT-PCR for all three fragments of PNRSV using tenfold serial dilutions of RNA. Lanes 1–6: 10^0^ (400 ng)-10^–5^ (0.004 ng) serial dilutions. Lane M: 1 kb DNA ladder (RTU, GeneDireX, Taiwan).

In one-step RT-PCR, RNA1 was identified up to 10^–2^ (4 ng), RNA2 up to 10^–4^ (0.04 ng), and RNA3 up to 10^–3^ (0.4 ng) dilution (Fig. 2a-c**,** Supplementary Fig. S2a–c). In one-step mRT-PCR, RNA1 had a detection limit up to 10^–3^ (0.4 ng), whereas RNA2 and RNA3 had a detection limit of up to 10^–4^ (0.04 ng) (Fig. 2d**,** Supplementary Fig. S2d).

### Sensitivity of two-step RT and mRT-PCR

**Figure 3:**
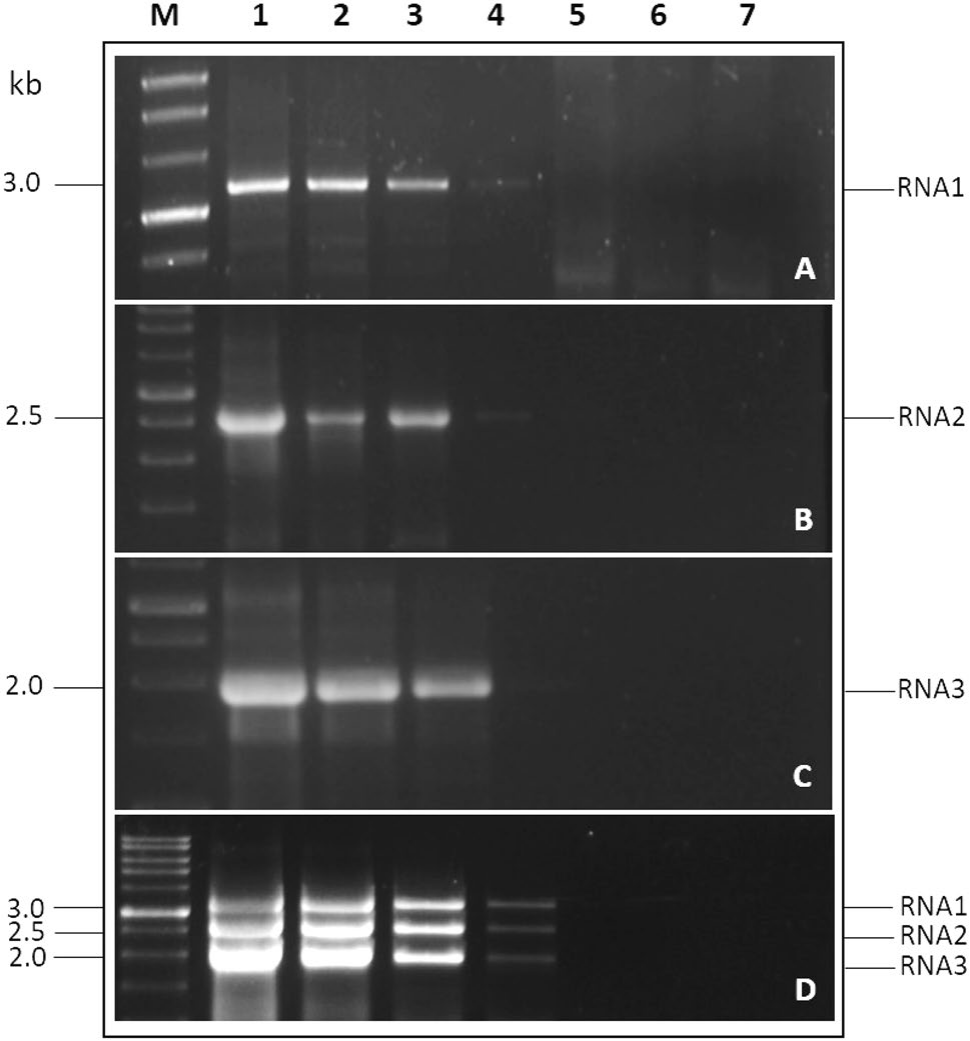
Sensitivity of two-step RT and mRT-PCR for all three fragments of PNRSV using tenfold serial dilutions of RNA. Lanes 1–6: 10^0^ (400 ng)-10^–5^ (0.004 ng) serial dilutions. Lane 7: water as a negative control. Lane M: 1 kb DNA ladder (RTU, GeneDireX, Taiwan).

A ten-fold serial dilution of RNA (10^0^–10^–5^) was used to assess the detection limit of two-step RT and mRT-PCR. In both two-step RT-PCR and mRT-PCR, RNA1, RNA2, and RNA3 could be identified clearly up to 10^–3^ (0.4 ng) dilution in RNA dilution (Fig. 3a–d, Supplementary Fig. S3a–d). In RT-PCR and mRT-PCR, there were no significant changes in the detection limits of all three PNRSV RNA fragments.

### Characterization of the whole genome of PNRSV (Indian Isolate)

**Figure 4:**
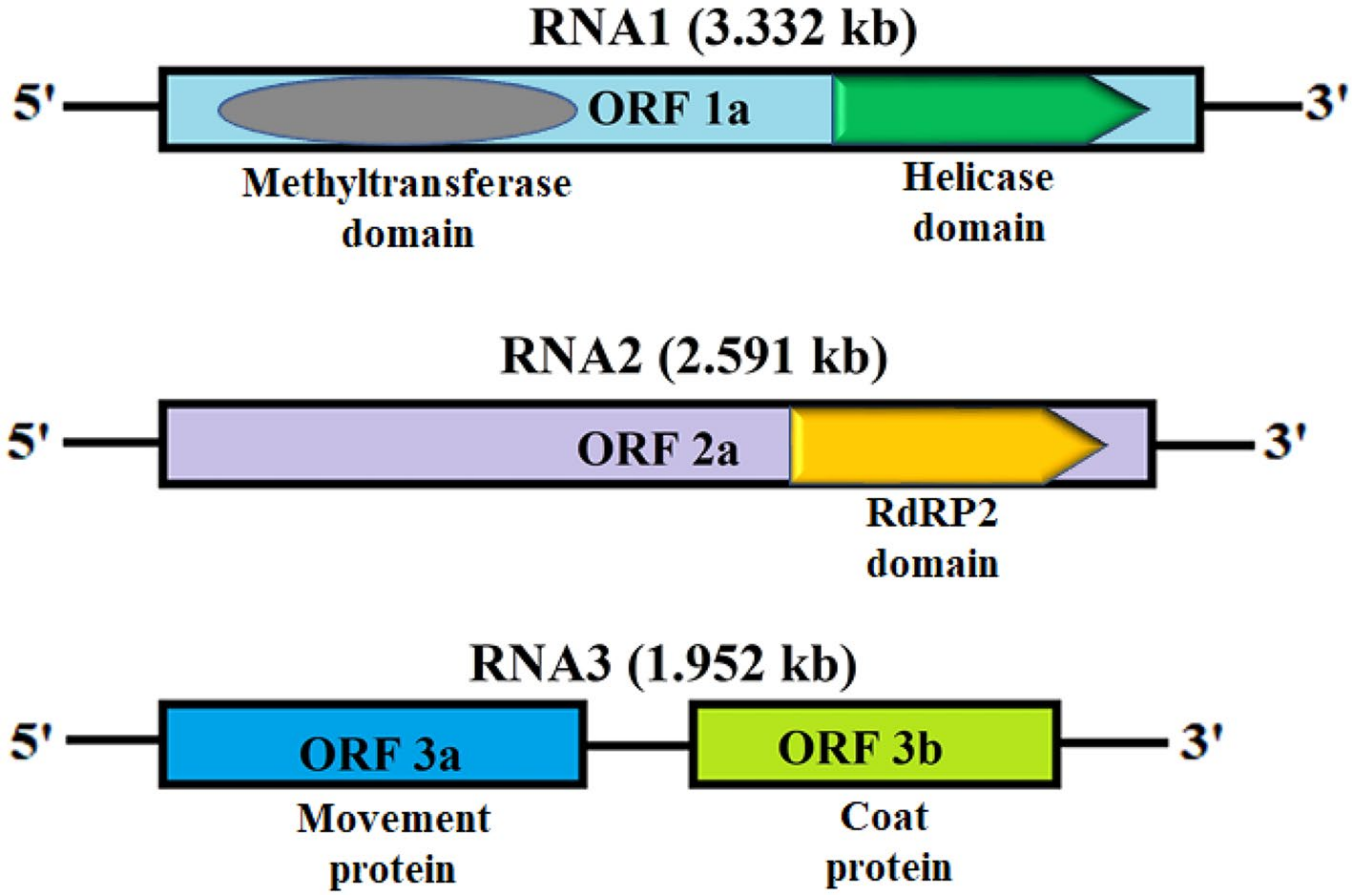
Diagrammatic illustration showing the characteristic domains and proteins of PNRSV RNA1, RNA2 and RNA3 encoded by their respective ORFs.

After sequencing analysis, RNA1, RNA2 and RNA3 nucleotide sequences of PNRSV obtained from infected apricot leaf samples were found to be 3.332 kb, 2.591 kb and 1.952 kb, respectively (Fig. 4). The obtained sequences were submitted to DDBJ with Accession Numbers viz. LC382449 (RNA1), LC382467 (RNA2) and LC382468 (RNA3). While the characteristic features of the genomic RNA sequences are mentioned in Table 2, RNA1 and RNA3 have an equal propensity of % GC content (45%), while RNA2 has 41% GC content. The open reading frames (ORFs) analysis and sequence alignment comparisons showed that ORF1a encodes Replicase1 (1045 aa), ORF2a Replicas2 (799 aa), ORF3a movement protein (283 aa), and ORF3b encodes the coat protein (226 aa) (Fig. 4 and Table 2). The RNA1 and RNA2 do not have any intergenic region (IR), while RNA3 has a short (77 nt) IR between movement protein (177–1028 nt) and coat protein (1104–1784 nt) ORFs of the PNRSV viruses.

**Table 2:** Characteristic features of RNA1, RNA2 and RNA3 of PNRSV.

Characteristic features	RNA1	RNA2	RNA3	
Accession No	LC382449	LC382467	LC382468	
Size (nts)	3332	2591	1952	
GC content (%)	45.62	41.99	45.90	
ORFs encoded proteins	Replicase P1 (RNA dependent RNA polymerase 1)	Replicase P2 (RNA dependent RNA polymerase 2)	Movement protein	Coat protein
Protein size amino acids (aa)	1045	799	283	226
Theoretical Molecular weight (kDa)	117	91	31	25
Total number of negatively charged residues (Asp + Glu)	134	115	37	22
Total number of positively charged residues (Arg + Lys)	137	80	38	28
Aliphatic index	84.51	82.83	92.90	86.55
Grand average of hydropathicity (GRAVY)	− 0.283	− 0.244	− 0.248	− 0.353
Theoretical isoelectric point (pI)	7.90	5.07	7.61	9.20
Start-stop codon (nt)	30–3167	27–2426	177–1028	1104–1784

### Sequence demarcation tool (SDT) analysis

**Figure 5:**
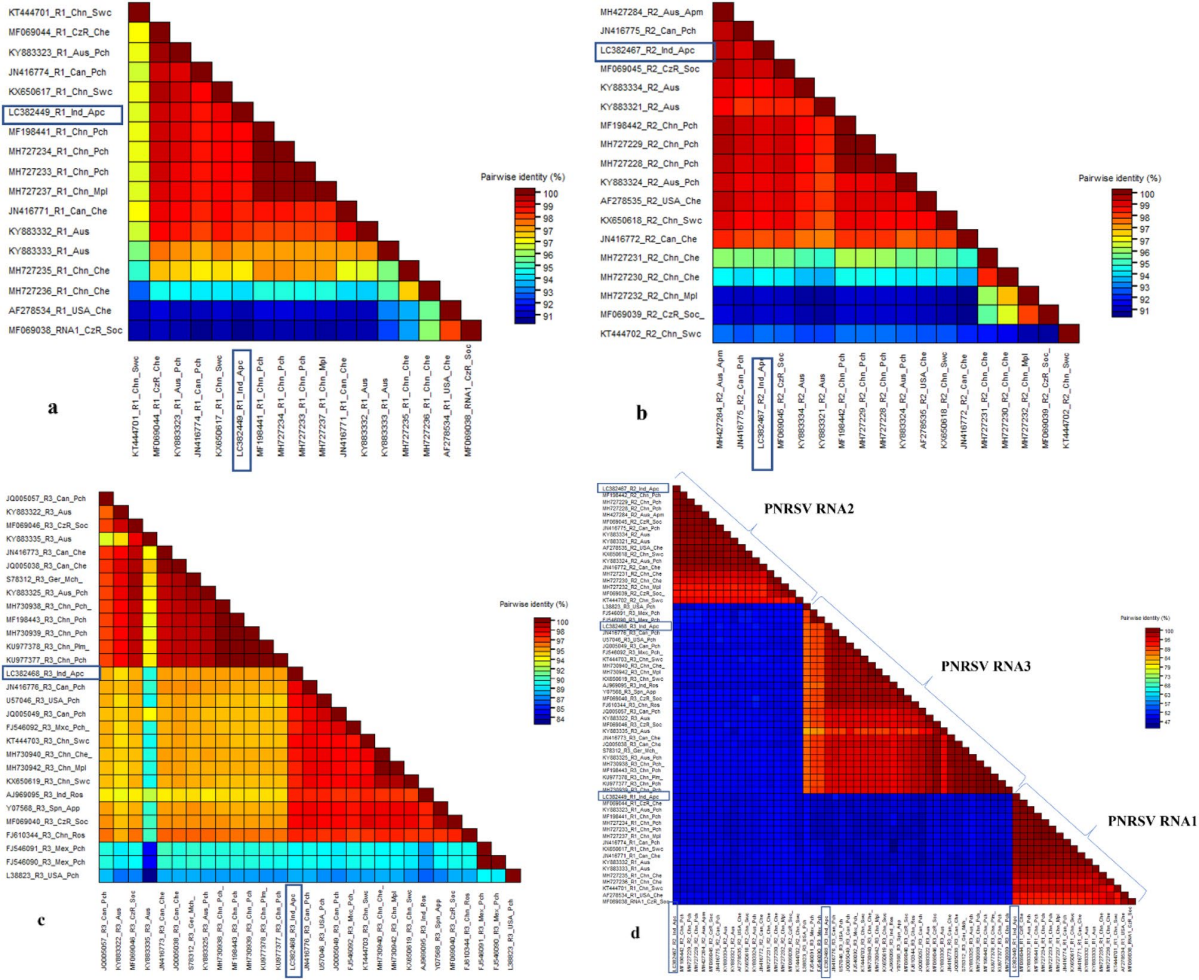
Sequence Demarcation Tool analysis displays the percentage pair-wise identity of Indian isolate of (**a**) RNA1, (**b**) RNA2, (**c**) RNA3 and (**d**) combination of RNA1, 2 and 3 sequences with other RNA1, 2 and 3 sequences of PNRSV from different geographical boundaries.

The SDT (version 1.2) analysis displayed the percentage pair-wise identity of understudy Indian isolate of PNRSV RNA1 sequence with other RNA1 sequences from different geographical boundaries, which varies from 91 to 99% (Fig. 5a). The Indian isolate showed nucleotide identity of 99% with RNA1 of the Czech Republic and Australian isolates obtained from cherry and peach respectively. Likewise, 98% of nucleotide identities were exhibited with eight other genomic RNA1 sequences, Candian Peach, Chinese Sweet cherry, Chinese Peach, Chinese Myrobalan plum, Canada Cherry and Australian cherry, respectively. The minimum pair-wise identity of 91% was shown with two PNRSV RNA1 sequences of Cherry and Sour Cherry submitted from the USA and Chez Republic (Fig. 5a).

Similarly, the SDT analysis unveiled that PNRSV genomic RNA2 under study showed a maximum nucleotide identity of 99% with eight other RNA2 sequences from different geographical locations and host species viz., Australian cherry, Canadian Peach, Czech Republic Sour cherry, China Peach, Australian Peach and USA Cherry. While the sequences China_ Myrobalan plum, and Czech Republic Sour Cherry showed a minimum pair-wise identity of 91% with the India Apricot understudy (Fig. 5b).

Likewise, The SDT analysis showed the percentage pair-wise identity of PNRSV RNA3 understudy with other RNA3 sequences from different geographical locations, which varies from 87 to 98% (Fig. 5c). The Indian isolate shared maximum nucleotide identity of 98% with twelve other PNRSV RNA3 sequences- Canadian Peach, USA Peach, Canadian Peach, Mexican Peach, China Sweet cherry, China Cherry, China Myrobalan plum, China Sweet cherry, Indian Rose, Spanish Apple, Czech Republic Sour cherry and, China Rose respectively (Fig. 5c). While one sequence, USA Peach, showed a minimum pair-wise identity of 87% with the India Apricot sequence understudy (Fig. 5c). This SDT analysis shows that similarities are higher between groups than among groups of RNA 1, RNA2 and RNA3 genomes when the three genomes were concatenated. Figure 5d clearly shows that the three genomic RNA sequences show a significant nucleotide identity among them.

### Recombination detection analysis

**Figure 6:**
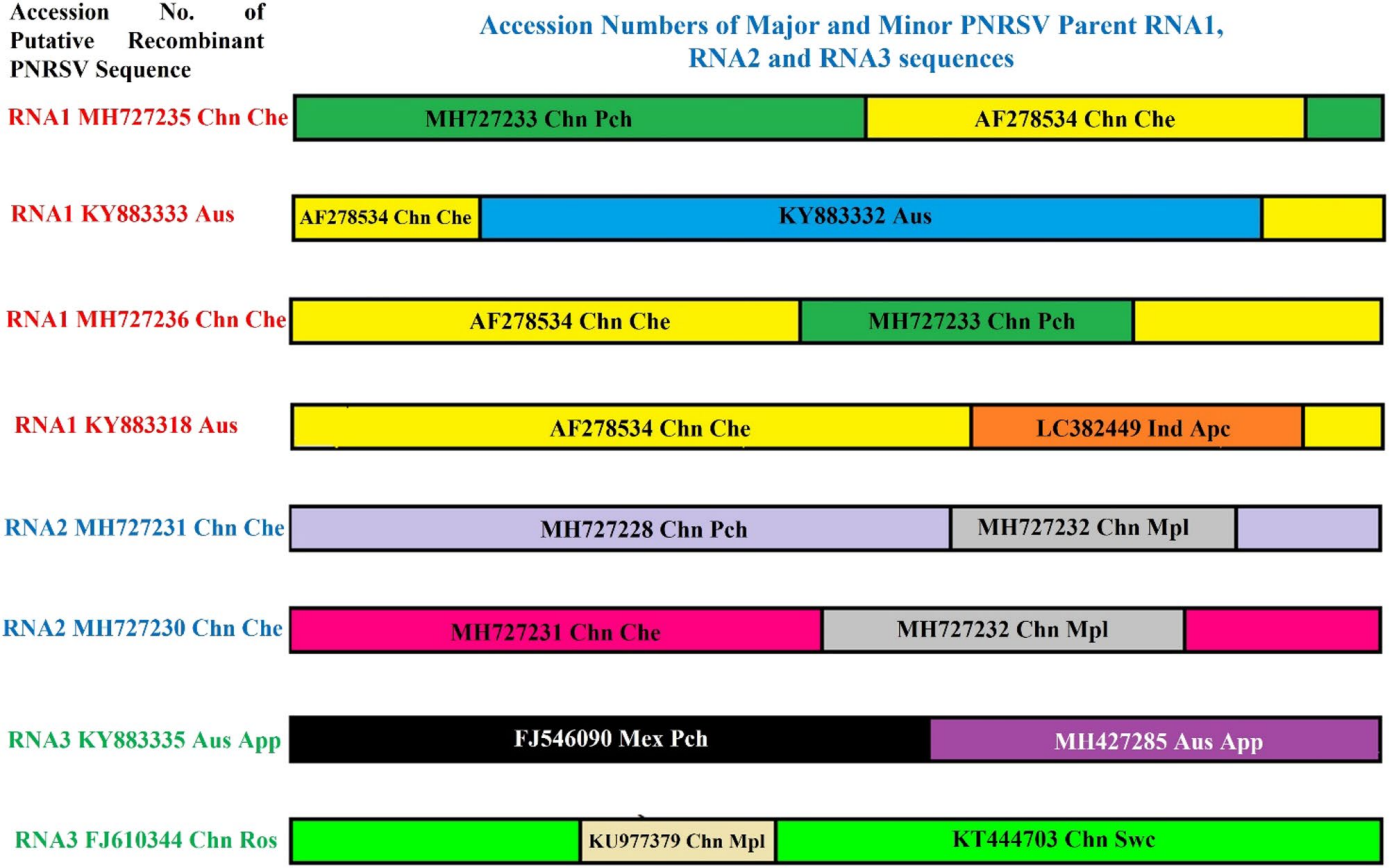
Recombination detection analysis of various isolates of PNRSV.

The RDP version 4.97 has detected twelve recombination signals in fourteen unique events in PNRSV RNA1 (Table 3). The PNRSV RNA1 sequence has GenBank Acc. No. MH727235 (China) was found most potential recombinant sequence isolated from cheery, which was detected by six methods (GENECONV, BootScan, MaxChi, Chimaera, Siscan and 3Seq) of RDP version 4.97. Compared to PNRSV RNA1 understudy, the other RNA1 sequences MH727235, MH727236, KY883318, and KY883333 may be recombinant (Table 3). The recombinant sequence MH727235 may be potentially originated due to recombination between MH727233 (major) and AF278534 (minor) sequences (Fig. 6). Similarly, MH727236 may be originated by the recombination of AF278534 (major) and MH727235 (minor) sequences. The Australian recombinant sequence KY883333 may be evolved by the recombination of KY883332 (major) and AF278534 (minor) sequences (Fig. 6). Meanwhile, RDP version 4.97 identified the LC382449 (India, Apricot) RNA1 sequence as a minor parent for the KY883318 (ApMV, Australia) RNA1 sequence, although with an exceptionally low likelihood of RDP, GENECONV and 3Seq methods (Table 3 and Fig. 6). Interestingly, it was found that AF278534 is acting as a major parent of two recombinant sequences- MH727236 and KY883318: meanwhile minor parent for MH727235 and KY883333 (Fig. 6). Overall, the PNRSV RNA1 understudy (LC382449) did not exhibit any strong sign of recombinant origin assisted as a minor parent.

**Table 3:** Analysis of recombination events in PNRSV RNA1, RNA2 and RNA3 sequences under study compared with other genomic RNA1, RNA2 and RNA3 sequences from different geographical locations worldwide.

Genomic Segment	Recombinant (Accession No.)	Major parent	Minor parent	Region (nt) derived from major parent	Region (nt) derived from minor parent	RDP	Geneconv	BootScan	MaxChi	Chimaera	SiScan	3Seq
RNA1	MH727235 (China, Cherry)	MH727233 (China, Peach)	AF278534 (US, Cherry)	1–1297 and 2119–3332	1298–2118	1.166 × 10^–27^	7.070 × 10^–24^	8.284 × 10^–21^	3.879 × 10^–19^	1.406 × 10^–17^	1.047 × 10^–20^	4.041 × 10^–14^
	MH727235 (China, Cherry)	MH727233 (China, Peach)	AF278534 (US, Cherry)	1–1297 and 2119–3332	1298–2118	1.566 × 10^–22^	7.061 × 10^–24^	8.284 × 10^–21^	3.879 × 10^–19^	1.406 × 10^–17^	1.047 × 10^–20^	4.041 × 10^–14^
	KY883333 (Australia)	KY883332 (Australia)	AF278534 (USA, Cherry)	855–3404	1–854 and 3405–3463	2.388 × 10^–34^	3.578 × 10^–32^	1.303 × 10^–19^	4.414 × 10^–15^	4.181 × 10^–15^	1.240 × 10^–18^	6.083 × 10^–40^
	MH727236 (China, Cherry)	AF278534 (USA, Cherry)	MH727233 (China, Peach)	1–2177 and 3407–3463	2178–3406	1.769 × 10^–27^	1.961 × 10^–29^	9.296 × 10^–25^	3.728 × 10^–21^	6.723 × 10^–21^	3.999 × 10^–28^	9.426 × 10^–43^
	KY883318 (Apple mosaic virus, Australia)	AF278534 (USA, Cherry)	LC382449 (India, Apricot )	1–2551 and 2703–3463	2552–2702	5.616 × 10^–09^	2.605 × 10^–08^	–	–	–	–	5.308 × 10^–04^
RNA2	MH727231 (China, Cherry)	JN416722 (China, cherry)	MH727228 (China, Peach)	1327–2573	1–1326, 2574–2591	1.728 × 10–07	2.733 × 10–07	–	2.133 × 10–06	4.635 × 10–05	–	2.670 × 10–06
	MH727231 (China, Cherry)	MH727228 (China, Peach)	MH727232 (China, Mpl)	1–1297 and 2119–3332	1298–2118	–	3.020 × 10–17	–	2.483 × 10–19	3.026 × 10–23	–	6.409 × 10–52
	MH727230 (China, Cherry)	MH727231 (China, Cherry)	MH727232 (China, Mpl)	1–883 and 1350–2591	884–1349	–	8.456 × 10^–12^	–	4.149 × 10^–10^	4.110 × 10^–10^	–	5.263 × 10^–22^
	MH727231 (China, Cherry)	MH727228 (China, Peach)	MH727232 (China, Mpl)	1–1349 and 2541–2591	1350–2540	–	3.020 × 10^–17^	5.153 × 10^–26^	1.121 × 10^–18^	3.026 × 10^–23^	–	8.409 × 10^–52^
	MH727230 (China, Cherry)	MH727231 (China, Cherry)	MH727232 (China, Mpl)	1–883 and 1350–2591	884–1349	–	8.453 × 10^–12^	4.598 × 10^–14^	4.149 × 10^–10^	4.110 × 10^–10^	–	5.308 × 10^–04^
	MH727231 (China, Cherry)	MH727232 (China, Mpl)	MH727228 (China, Peach)	1351–2539	1–1350 and 2540–2591	–	1.280 × 10^–21^	–	4.959 × 10^–20^	6.032 × 10^–17^	–	–
	MH727230 (China, Cherry)	AF278535 (USA, Cherry)	MH727232 (China, Mpl)	1–883 and 1350–2591	884–1349	–	2.314 × 10^–10^	–	2.382 × 10^–10^	2.538 × 10^–10^	–	–
	MH727232 (China, Mpl)	MH727228 (China, Peach)	MH727231 (China, Cherry)	1351- 2539	1- 1350 and 2540–2591	–	1.949 × 10^–18^	–	3.763 × 10^–19^	–	2.156 × 10^–32^	1.413 × 10^–54^
	MH727230 (China, Cherry)	KX650618 (China, Sweet Cherry)	MH727232 (China, Mpl)	1–883 and 1350–2591	884–1349	–	2.501 × 10^–11^	1.508 × 10^–13^	7.718 × 10^–10^	7.497 × 10^–10^	1.564 × 10^–14^	2.500 × 10^–21^
	MH727230 (China, Cherry)	MH727228 (China, Peach)	JN416772 (Canada, Cherry)	1–1350 and 2541- 2591	1351- 2540	–	–	–	–	–	2.165 × 10^–19^	1.888 × 10^–02^
RNA3	KY883335 (Australia, Apple)	FJ546090 (Mexican, peach)	MH427285 (Australian Apple)	1–203	1982–2073	–	2.351 × 10^–13^	1.556 × 10^–14^	8.452 × 10^–15^	–	4.722 × 10^–34^	4.583 × 10^–41^
	FJ610344 (China, Rose)	KT444703 (China, Sweet Cherry)	KU977379 (China, Myrobalan plum)	1–917 and 1174–2106	918–1173	–	2.247 × 10^–02^	2.972 × 10^–04^	1.102 × 10^–05^	1.339 × 10^–03^	4.462 × 10^–07^	2.594 × 10^–02^
	KU977377 (China, Peach)	JN416773 (Canadian Cherry)	FJ546090 (Mexican Peach)	1–1940	1941–2073	–	2.684 × 10^–02^	–	–	–	–	–
	FJ546092 (Mexican Peach)	LC382468 (Under study)	FJ546090 (Mexican Peach)	37–1940	1–36 and 2011–2073	–	–	1.093 × 10^–02^	–	–	–	–
	U57046 (USA, Peach)	KT444703 (China, Sweet cherry)	JQ005057 (Canadian Peach)	270–2003	1–269	–	4.011 × 10^–02^	–	–	–	–	− ^c^

Compared to the PNRSV RNA2 understudy, RDP version 4.97 detects fifteen recombination signals in twelve unique events in other PNRSV RNA2 sequences from different geographical locations (Table 3). The PNRSV RNA2 sequence had GenBank Acc. No. MH727231 (China, cherry) and MH727230 (China, cherry) were found potential recombinant sequence isolated from cheery, which was detected by five and four methods of RDP version 4.97 viz., RDP, GENECONV, BootScan, MaxChi, and Chimaera (Table 3). The RNA2 sequence with Acc. No. MH727231 (China, cherry) is most probably originated due to the recombination between MH727228 (China, Peach) and MH727232 (China, Mpl), as a major and minor parent, respectively (Fig. 6). Similarly, the PNRSV RNA2 sequence having Acc. No. MH727230 (China, Cherry) was found to be another potential recombinant generated by the recombination of MH727231 (China, Cherry) and MH727232 (China, Myrobalan plum) as a major and minor parent, respectively (Fig. 6). Meanwhile the lowest p-values of 3Seq, BootScan, Chimaera, MaxChi, and GENCONV of 8.409 × 10^–52^, 5.153 × 10^–26^, 3.026 × 10^–23^, 1.121 × 10^–18^ and 3.020 × 10^–17^ respectively reflects highest probability of MH727231 (China, cherry) is derived due to recombination between MH727228 (China, Peach) and MH727232 (China, Myrobalan plum) sequences (Table 3). There was no evidence of a recombination event in the PNRSV RNA2 understudy.

RDP version 4.97 reveals seventeen recombination signals in seventeen unique events in various PNRSV RNA3 sequences from different geographical regions, compared to the PNRSV RNA3 (LC382468, India, Apricot) understudy (Table 3). The PNRSV RNA3 sequence has GenBank Acc. No. FJ610344 (China) and KY883335 (Australia) were found to be the most potential recombinant sequence isolated from peach and rose, respectively. They were detected by six and five methods of RDP version 4.97 viz., GENECONV, BootScan, MaxChi, Chimaera, SiScan and 3Seq, respectively (Table 3). The RNA3 sequence with Acc. No. KY883335 (Australia) was originated due to the recombination between FJ546090 (Mexican, peach) and MH427285 (Australian, Apple) as a major and minor parent, respectively (Fig. 6). Similarly, the PNRSV RNA3 sequence with Acc. No. FJ610344 (China, Rose) was found to be another potential recombinant originating by the recombination of KT444703 (China, Sweet cherry) and KU977379 (China, Myrobalan plum) as a major and minor parent, respectively (Fig. 6). But the lowest p-values of 3Seq, SiScan, MaxChi, BootScan, and GENCONV, of 4.583 × 10^–41^, 4.722 × 10^–34^, 8.452 × 10^–15^, 1.556 × 10^–14^ , and 2.351 × 10^–13^ respectively reflects that KY883335 (Australia) is strongly recombination RNA3 sequence (Table 3). In this analysis, we found that the PNRSV RNA3 sequence under study acts as a major parent for the origination of a putative recombinant FJ546092 (Mexican, Peach) RNA3 sequence, but the chances of occurrence are low because it is detected by only one RDP method viz., GENCONV having p-value 1.093 × 10^–02^.

### Phylogenetic analysis

**Figure 7:**
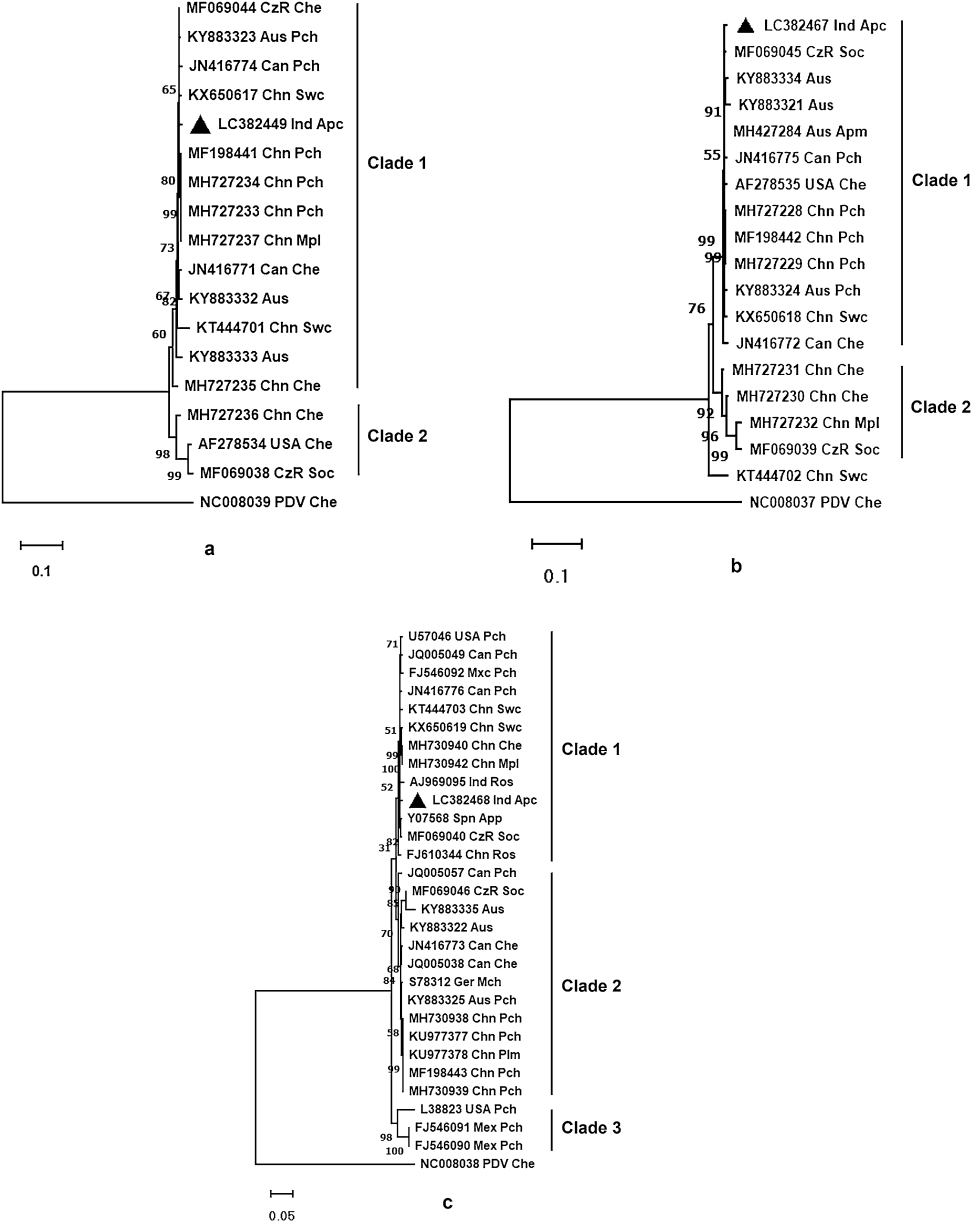
Phylogenetic trees (**a**, **b**, **c**) constructed with the Neighbor-Joining methodusing the full nucleotide sequences of RNA 1, 2 and 3 from prunus necrotic ringspot virus (PNRSV) isolates and prune dwarf virus (PDV) as the outgroup. In total, 17 (RNA1), 18 (RNA2) and 28 (RNA3) complete putative sequences were shortlisted for this study. MEGA11 were used to build pairwise, multiple sequence alignments, and visualize the tree. The accession number, country of origin, and natural host of each isolate are all listed on the terminal location of the branch. The black triangle represents the Indian isolate under study.

The phylogenetic NJ analysis revealed PNRSV RNA1 has two major clades, with clade 1 having the RNA1 Indian isolates from apricot, which is the closest relative of the PNRSV sequences isolated from the sweet cheery of China (Fig. 7a). This study also revealed that clade 1 has phylogenetically most similar RNA1 sequences from diverse origins, including China, Australia, the Czech Republic, Canada, and India (Fig. 7a). The clade2 has three less similar sequences belonging to China, the USA and the Czech Republic. In the phylogenetic analysis, the prune dwarf virus RNA1 is found on a distinct branch as an out-group.

Similarly, the phylogenetic NJ analysis for PNRSV RNA2 Indian isolates from apricot revealed the most closely related phylogenetic relationship with PNRSV sequences isolated from Czech Republic sour cherry (Fig. 7b). The PNRSV RNA2 sequences were similarly found to be divided into two clades. Sequences from China, the United States, Australia, the Czech Republic, Canada, and India make up the first clade of highly similar sequences (Fig. 7b). Furthermore, the PDV RNA2, shown on a distinct branch as an out-group, is close to the second clade of less comparable sequences (from China and the Czech Republic).

Meanwhile, the NJ phylogenetic analysis for the PNRSV RNA3 of Indian isolates from apricot under study clearly showed that it is phylogenetically closest to the already reported PNRSV genomic RNA3 sequence isolated from the Indian rose (Fig. 7c). The complete genomic RNA3 sequences are categorized into three clades, and the two bigger clades contain thirteen sequences each. The third and smallest clade contains three sequences close to the PDV RNA3 shown on a separate branch as an out-group. The Indian isolate comes under the first clade, with sequences from diverse origins like India, China, Mexico, Canada, the Czech Republic, Spain, and the USA.

## Discussion

PNRSV is a plant virus that infects a wide range of *Prunus* species, resulting in significant economic losses^1^. Based on CP gene sequences, Noorani et al.^9^ investigated the molecular variability of PNRSV associated with apricot trees growing in the Union Territory of Jammu and Kashmir region of India. To date, however, no whole genomic sequence of an Indian PNRSV isolate has been sequenced. Here, we amplified and characterized the first whole genome of an Indian PNRSV apricot isolate designated as ‘Acot’.

The whole genome of PNRSV, including RNA1, RNA2, and RNA3, was successfully amplified using one-step RT-PCR and mRT-PCR, as well as two-step RT-PCR and mRT-PCR. The results showed that one-step mRT-PCR is more sensitive than two-step mRT-PCR. One-step mRT-PCR had a detection limit of 10^–4^ (0.04 ng) for RNA2 and RNA3, while two-step mRT-PCR had a detection limit of only 10^–3^ (0.1 ng) for RNA1, RNA2, and RNA3 (0.4 ng). In one-step RT-PCR, RNA1 was identified up to 10^–2^ (4 ng), RNA2 up to 10^–4^ (0.04 ng), and RNA3 up to 10^–3^ (0.4 ng) dilution. In this case, the detection limit is different for each fragment of RNA, even though the concentration of total RNA is the same in each reaction of the one-step RT-PCR. The variance in detection limits may be attributed to the number of copies of each RNA in the given sample. The copy number of the RNA 1, 2, and 3 transcripts may differ at different stages of the virus's lifecycle in the host tissue.

Although the pathological effect of PNRSV infection on the host plants (*Prunus armeniaca, P. domestica, P. persica, P. nucipersica, P. avium, P. dulcis, P. cerasifera)* and the necrotic ringspot diseases progression is still in infancy. PNRSV is more widely distributed and affects several plant species but remains undiagnosed because symptoms associated with PNRSV are similar to other Ilarviruses viz., American plum line pattern virus (APLPV), apple mosaic virus (ApMV), prune dwarf virus (PDV) in prunus hosts. Therefore, if PNRSV remains undiagnosed in its initial stage of infection, it causes severe yield losses.

SDT analysis revealed that the RNA1 and RNA2 of the Indian isolate of PNRSV had 91–99% sequence identity, while the RNA3 had 87–98% sequence identity at the nucleotide level. Our results reveal the presence of genetic diversity within the PNRSV species from different geographical locations of the world. Interestingly, it was also found that there is high host diversity for PNRSV which might affect its pathogenicity and severity. Therefore, there are clear implications for its early detection and accurate molecular diagnostics for best disease management practices.

In this study, some potential recombination events in the RNA1, RNA2 and RNA3 sequences of different isolates from diverse geographical regions of the world are detected. The lack of strong recombination signals in the RNA1, RNA2 and RNA3 sequences under study suggests that the PNRSV genome (apricot) of Indian isolate is purely non-recombination genome or may be a cold spot of recombination for only a few sequences, for instance, RNA1 (KY883318) and RNA2 (FJ546092). The non-existence of recombinant PNRSV isolates in India could be attributed to the lack of genomic sequences of Ilarvirus from the Indian subcontinent. Recombination signals in PNRSV from China are due to many available NCBI accessions and multiple isolates of PNRSV.

This study performed phylogenetic reconstruction of complete genomic RNA1, RNA2 and RNA3 sequences, and the evolutionary relationship between different PNRSV virus isolates was calculated. The phylogenetic analysis revealed two distinct clades based on the complete nucleotide sequences of RNA1 and RNA2, whereas three clades were based on RNA3. The understudied Indian isolate of PNRSV is a member of clade 1 in all three trees. Recently, Kinoti et al.^31^ proposed two phylogenetic groups for each of RNA1 (PG1 and PG2) and RNA2 (PG1 and PG2) and three phylogenetic groups for RNA3 (PG1, PG2, and PG3) based on methyltransferase (MT), RNA dependent RNA polymerase (RdRp) and the CP gene segments of PNRSV. Earlier, PNRSV had been classified into three phylogroups (PV32, PV96, and PE5:^32–34^) and four phylogroups (PV32, PV96, PE5, and CH30:^8,32,33,35^) based on coat protein (CP) sequence. Based on RNA1, 2 and 3, the Indian PNRSV isolate is closely related to the Chinese sweet cherry isolate (Fig. 7a), the Czech Republic sour cherry isolate and (Fig. 7b) and the Indian rose isolate (Fig. 7c).

## Conclusion

In this study, we have amplified and characterized the whole genome of PNRSV from apricot employing one and two-step RT-PCR and mRT-PCR. One-step mRT-PCR was shown to be more sensitive than two-step mRT-PCR. In one-step mRT-PCR, the detection limit for RNA2 and RNA3 was 10^–4^ (0.04 ng), whereas in two-step mRT-PCR, the detection limit for RNA1, RNA2, and RNA3 was only 10^–3^ (0.4 ng). During recombination analysis, we did not find any potential recombinants from the whole genome of an Indian isolate of PNRSV probably due to a scarcity of whole genome sequences of other Ilarvirus from the Indian subcontinent. Furthermore, the phylogenetic NJ analysis based on full length RNA1, 2, and 3 sequences demonstrated that the Indian isolate of PNRSV belongs to clade 1. In conclusion, this research will be useful for the simultaneous amplification of all three RNA fragments of PNRSV in a single step, which will further aid in the generation of infectious clones and thus be valuable for pathogenicity studies and the establishment of disease management strategies.

## Supplementary Information

Supplementary Information 1.

Supplementary Information 2.

## Data Availability

The datasets generated and/or analysed during the current study are available in the DDBJ (http://getentry.ddbj.nig.ac.jp/)/EMBL/GenBank repository under accession numbers LC382449 (http://getentry.ddbj.nig.ac.jp/getentry/na/LC382449/?format=flatfile&filetype=html&trace=true&show_suppressed=false&limit=10), LC382467 (http://getentry.ddbj.nig.ac.jp/getentry/na/LC382467/?format=flatfile&filetype=html&trace=true&show_suppressed=false&limit=10), LC382468 (http://getentry.ddbj.nig.ac.jp/getentry/na/LC382468/?format=flatfile&filetype=html&trace=true&show_suppressed=false&limit=10).
