# Supplementary material for: Whole genome characterization and diagnostics of prunus necrotic ringspot virus (PNRSV) infecting apricot in India
Source: Sci Rep. 2023 Mar 16;13:4393. doi: 10.1038/s41598-023-31172-z (PMC10020458; doi:10.1038/s41598-023-31172-z)
Supplement: Supplementary file 1 — Supplementary Information 1. [file 41598_2023_31172_MOESM1_ESM.docx]

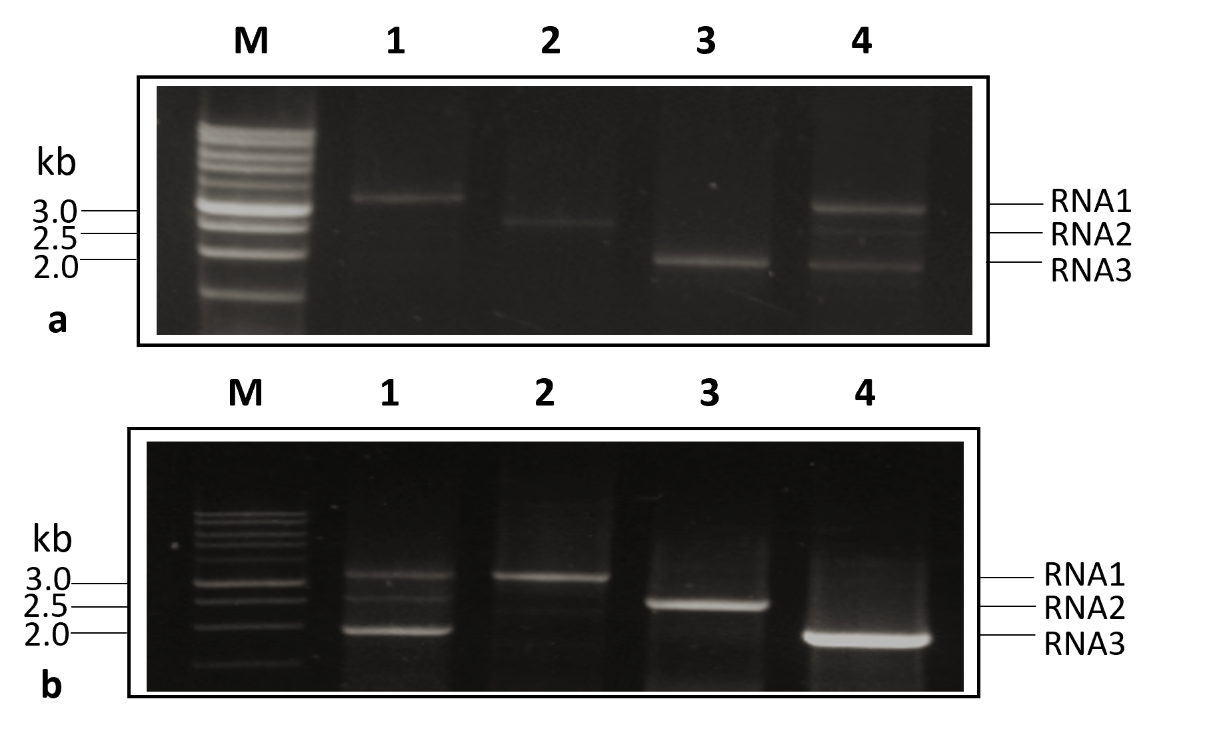


**Figure 1a-b (Cropped)**


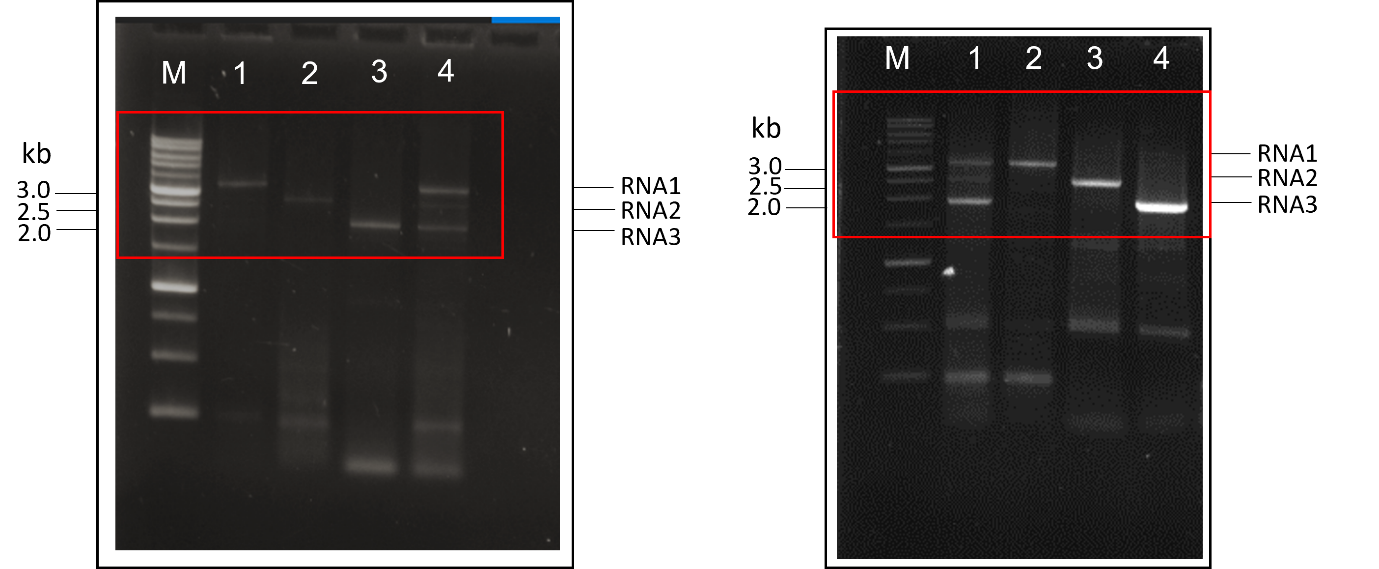


a b

**Supplementary Figure S1a-b (Uncropped)**

**Figure 1:** Amplification of whole genome of PNRSV by (a) one-step RT-PCR: Lane 1, 2 and 3 showing amplification of RNA 1, RNA 2 and RNA 3 respectively. Lane 4 is showing simultaneous amplification of RNA1, 2 and 3 by one-step mRT-PCR. (b) two-step RT-PCR: Lane 2, 3 and 4 showing amplification of RNA 1, RNA 2 and RNA 3 respectively. While Lane 1 shows simultaneous amplification of RNA 1, 2 and 3 by two-step mRT-PCR. Lane M showing 1 kb DNA ladder (RTU, GeneDireX).


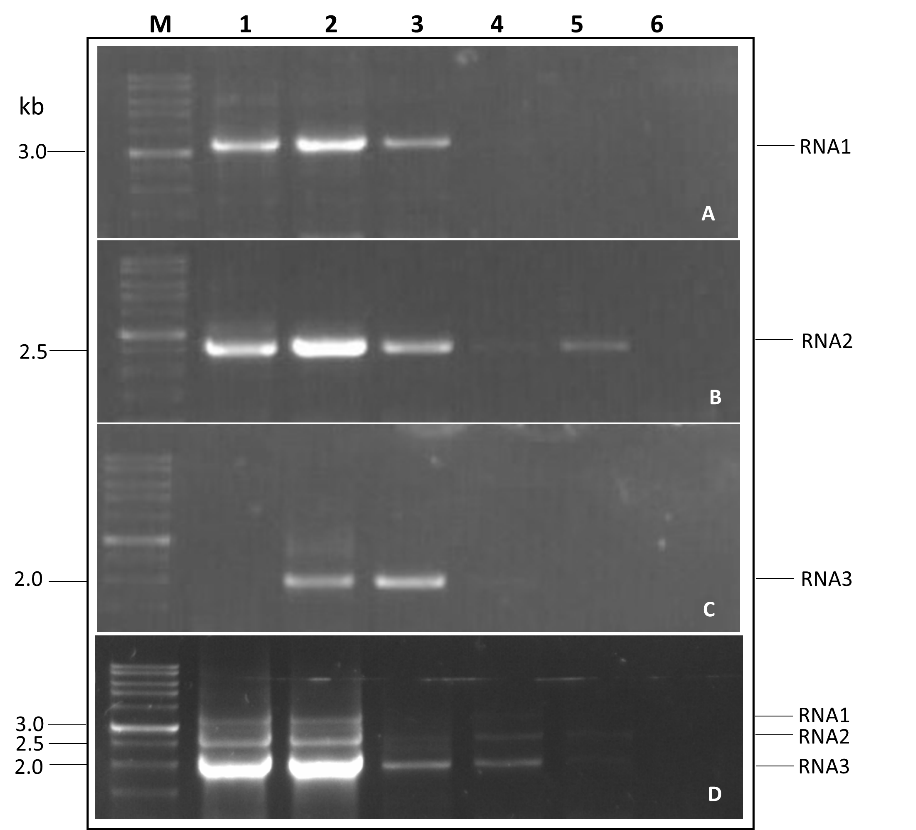


**Figure 2a-d (Cropped)**


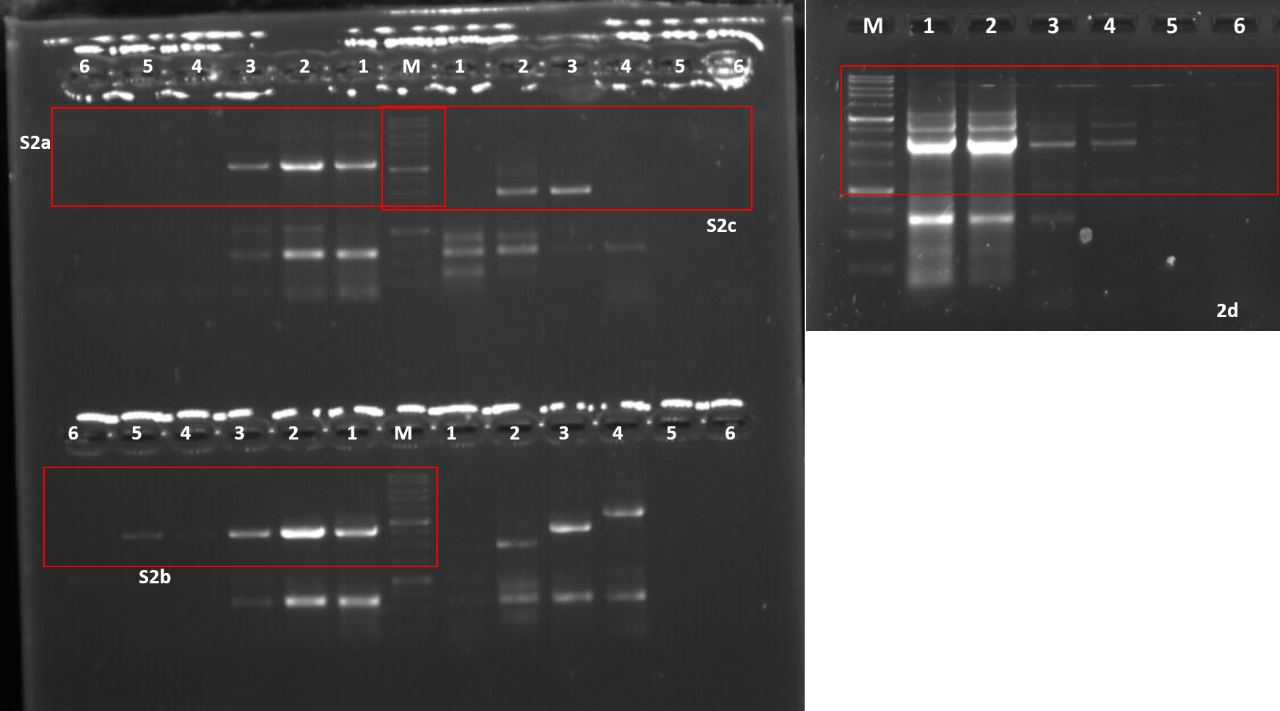


**Supplementary Figure S2a-d (Uncropped)**

**Figure 2.** Sensitivity of one-step RT and mRT-PCR for all three fragments of PNRSV using 10-fold serial dilutions of RNA. Lanes 1–6: 10^0^ (400 ng)-10^-5^ (0.004 ng) serial dilutions. Lane M: 1 kb DNA ladder. RNA1, 2 and 3 were detected up to (a) 10^-2^ (4 ng), (b) 10^-4^ (0.04 ng), (c) 10^-3^ (4 ng) dilution in RT-PCR. In one-step mRT-PCR, RNA1 had a detection limit up to (d) 10^-3^ (0.4 ng), whereas RNA2 and RNA3 had a detection limit of up to 10^-4^ (0.04 ng).


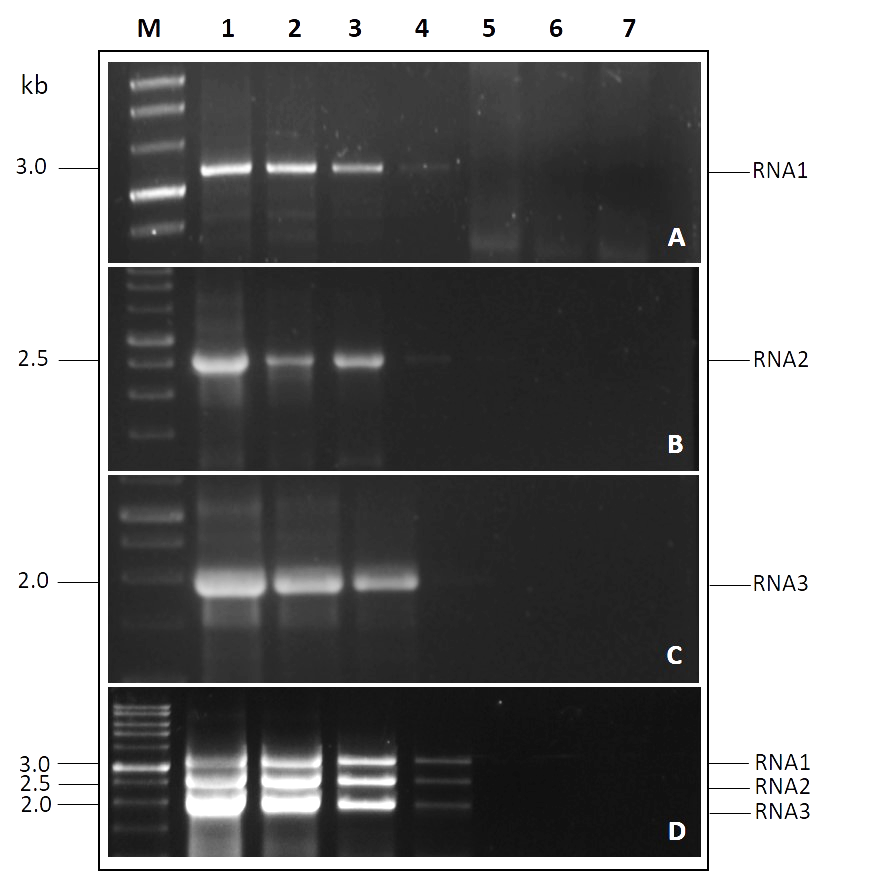


**Figure 3a-d (Cropped)**


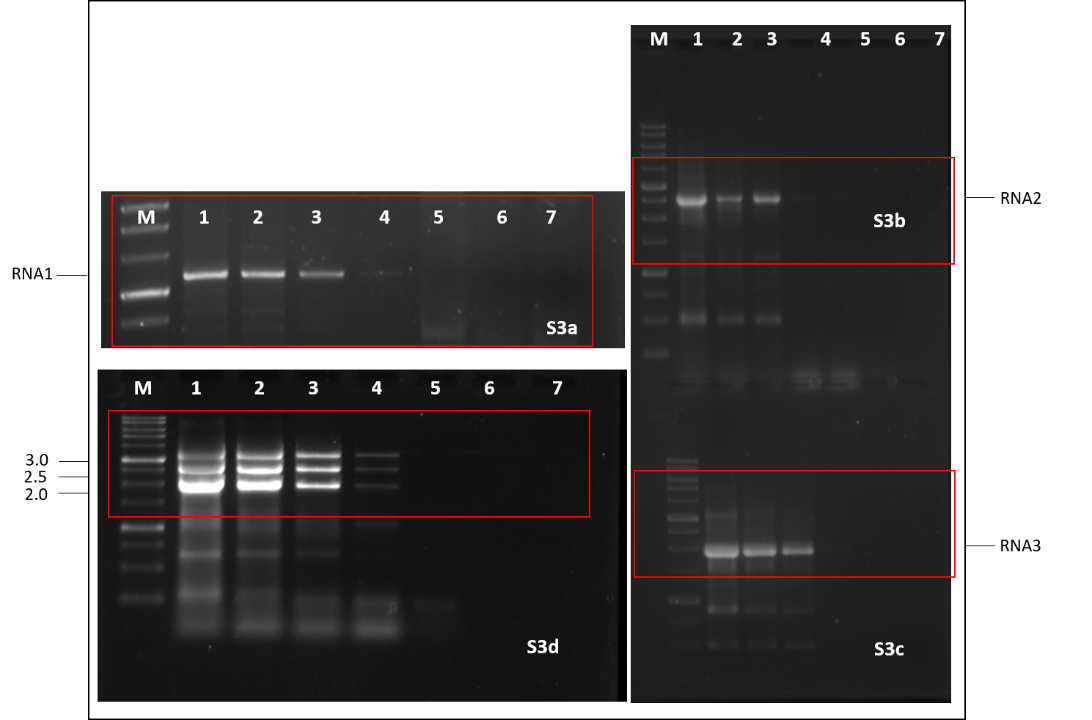


**Supplementary Figure 3a-d (Uncropped)**

**Figure 3.** Sensitivity of two-step RT and mRT-PCR for all three fragments of PNRSV using 10-fold serial dilutions of RNA. Lanes 1–6: 10^0^ (400 ng)-10^-5^ (0.004 ng) serial dilutions. Lane M: 1 kb DNA ladder. RNA1, 2 and 3 were detected up to 10^-3^ (0.4 ng) in RT-PCR (a, b, c). In two-step mRT-PCR, RNA1, 2 and 3 had a detection limit up to (d) 10^-3^ (0.4 ng). Lane 7: water as a negative control.
