# Supplementary material for: Whole genome characterization and diagnostics of prunus necrotic ringspot virus (PNRSV) infecting apricot in India
Source: Sci Rep. 2023 Mar 16;13:4393. doi: 10.1038/s41598-023-31172-z (PMC10020458; doi:10.1038/s41598-023-31172-z)
Supplement: Supplementary file 2 — Supplementary Information 2. [file 41598_2023_31172_MOESM2_ESM.docx]

**Supplementary Table 1.** Identification of Putative Regulatory Motifs of PNRSV RNA1, RNA2 and RNA3 encoded proteins (Replicase P1, Replicase P2, Movement and Coat proteins)

| **RNA 1, 2, and 3 encoded proteins** | **Major Putative Regulatory Motifs and Domains Identified by MotifFinder and motifscan program searching the** Pfam and NCBI-CDD | **Motif & Domain Name** | **E-value** (HMMER algorithm) | **Start-End positions** | **Major Functions** | **References** |
| --- | --- | --- | --- | --- | --- | --- |
| **Replicase1 protein** | RLLPALREALTVEAKFTRKLMDGVAGCGKTTKILNECKMCNDQPDLVLTSN | Mitochondrial ribosomal death-associated protein 3 (DAP3) | 0.26 | 735-785 | It induces [apoptosis](http://en.wikipedia.org/wiki/Apoptosis) and acts as a pro-apoptotic factor in the [mitochondrial matrix](http://en.wikipedia.org/wiki/Mitochondrial_matrix). This motif is very crucial for [mitochondrial](http://en.wikipedia.org/wiki/Mitochondrion) [biogenesis](http://en.wikipedia.org/wiki/Biogenesis). | Kissil et al., 1999; Berger et al., 2000 |
|  | AKFTRKLMDGVAGCGKTTKILNECKMCNDQPDLVLTSNRSSAMELREKLPGSQLLRSTRVRTSDSYLMNPKRPSSVRVIFDECFLQHAGCVYAAASLAGAEELVLFGDTKQIPFV | AAA 19 motif | 0.58 | 748-862 | AAA (ATPases Associated with diverse cellular Activities) motif is a critical part of many proteins that regulate several processes in DNA replication, protein disintegration, membrane fusion, microtubule spitting, peroxisome biosynthesis, signal transduction, and signal transduction energy-dependent remodelling or translocation of macromolecules. | Iyer et al., 2004; Lupas and Frickey 2004; Erzberger and Berger 2006; Hanson and Whiteheart 2005 |
|  | AEELVLFGDTKQIPFVSRIPHFRLKDHLVSADEKVMSN | Hexokinase motif | 0.94 | 847-884 | Hexokinase is a key enzyme in the ATP-dependent conversion of Aldo- and keto-hexose sugars like glucose, fructose, sorbitol, and glucosamine to hexose-6-phosphate (H6P). This process is the first step in several metabolic pathways. | [Griffin](https://pubmed.ncbi.nlm.nih.gov/?term=Griffin+LD&cauthor_id=1783373) et al., 1991 |
|  | SFALSAEQKSLLSRNFPGRDIHFAQRDSSSHSFAAAHRLLETDYIYKCFGTTEEAVIDLGGNFVSHIKQKRYNVHSCCPLLDDRDGARFTERLISLKTYLRTHKEERHEADYCECRFEECPRRADYVMAVHAVSDLPITDLCAALTKKGTKKMILSIMMDPNMLLRDVGEIPNFNVRWEIDRTEDIIRFDFIDAPCLGYQHKFSVLQQYLTTNAVIVGDKAAYRVERKSDFGGVFIVDITAVAGYKPGMVVGGTRSCAWSTLIRNKTVVHTVDGEDHWWYDVTRRSKILVDTKVLTKVLEASFRQFKPNVEPESMIQNIATMLSSSTNYTVINGVTLQAGESLPYGDYVAIAPTIYVRTKRMY | Viral Methyltransferase (at 5’ terminal) | 2.3e-68 - 2.5e-72 | 51-418 | This viral RNA methyltransferase domain is found in a wide range of ssRNA viruses of the Alphavirus superfamily, including alphaviruses and several other groups viz., Hordei-, Tobra-, Tobamo-, Bromo-, Clostero- and Caliciviruses. It forms the defining, unique feature of this superfamily. This guanine-7-methyltransferase Domain is involved in mRNA capping, which enhances its stability. As a result, many viruses that replicate in the cytoplasm produce their own methyltransferase. | [Rozanov *et al.*, 1992](https://www.ncbi.nlm.nih.gov/pmc/articles/PMC4635493/#jgv000249-Rozanov1); [Schluckebier](https://www.ncbi.nlm.nih.gov/pmc/articles/PMC4635493/#jgv000249-Schluckebier1)*[et al.](https://www.ncbi.nlm.nih.gov/pmc/articles/PMC4635493/#jgv000249-Schluckebier1)*[, 1995](https://www.ncbi.nlm.nih.gov/pmc/articles/PMC4635493/#jgv000249-Schluckebier1); [Martin & McMillan, 2002](https://www.ncbi.nlm.nih.gov/pmc/articles/PMC4635493/#jgv000249-Martin1); |
|  | LMDGVAGCGKTTKILNECKMCNDQPDL VLTSNRSSAMELREKLPGSQLLRSTRVRTSDSYLMNPKRPSSVRVIFDECFLQHAGCVYAAASLAGAEELVLFGDTKQIPFVSRIPHFRLKDHLVSADEKVMSNLTYRCPADATMALSKWFYRRNVKTANTTLRSMSVKPIVSVSQIDRNFDLYMTHTQAEKHTLIASGVVPRDKVFTSAEAQGKTEGRAALVRLSRTSMSLFTGKDPLMGPCHSLVALSRFKRQFVYFT | RNA helicase1 (at 3’ terminal) | 7.7e-48 - 8.6e-52 | 753-1010 | Viral RNA helicase1 belongs to Superfamily1 and plays multiple helicases and NTPase activity roles at different stages of viral RNA replication. Their roles have been dissected and proved by mutational analysis. | Fernandez A et al., 1995; Kadaré and Haenni 1997 ; Cordin O et al., 2006 ; Pyle AM. 2008 |
| **Replicase2 protein** | TSDINLEIDRASIDMSVFNDWTPKSGRLNGLFQTGNISKRVPTFREASLAIKKRNLNVPDLQQVLHEDDEARFIANKFINTVIDPNKLAQFPGYISEGEMGYFNKYLSGKAVPDDAFVDPCALVSMDKYRHMIKTQLKPVEDTSQLFERPLAATITYHDKGKVMSTSPIFLMMCNRLLLCLNDKISIPSGKHHQLFSLDPFAFEMTKEFKEIDFSKFDKSQQRLHHLIQFHIFTALGAPKDFLDMWFGSHEISHIRDGPCGIGFSVNYQRRTGDACTYLGNTIITLSTLAYMYDLLDPNVTFVIASGDDSLIGSIKPLDRSEEFKFVTLFNFEAKFPHNQPFVCSKFLCLLPTVSGGKKVLAVPNALKLNIKLGVKDLAPCVFDAWYKSWLDLIWYFDNYLVVSTMKDYISHRYLRRQTCYQEGAMLAYRTIFSSKEKC | RNA-dependent RNA polymerase ([RdRP2](https://www.genome.jp/dbget-bin/www_bget?pf:RdRP_2)) domain (at extreme 3’ terminal) | 1.3e-129 - 7.1e-134 | 311-751 | The Replicase2 holds domains of 2A protein from bromoviruses, putative RNA-dependent RNA polymerase from tobamoviruses and Non-structural polyprotein from togaviruses. | [[R Quadt](https://pubmed.ncbi.nlm.nih.gov/?term=Quadt+R&cauthor_id=8434011)  et al., 1993; Smirnyagina](https://pubmed.ncbi.nlm.nih.gov/?term=Smirnyagina+E&cauthor_id=8676500) et al., 1996 |
|  | KRVPTFREASLAIKKRNL | Bipartite nuclear localization signal motif | 2.1e+04 | 349-366 | At the cellular level, the relocalization of host proteins via interactions with viral components is an emerging mechanism through which viruses affect host physiology. The ability of the TMV replicase to block the nuclear localization of specific Aux/IAA proteins correlates with the transcriptional alterations of auxin-responsive genes and the display of disease symptoms. | [Padmanabhan](https://www.ncbi.nlm.nih.gov/pubmed/?term=Padmanabhan%20MS%5BAuthor%5D&cauthor=true&cauthor_uid=15681455) et al., 2005 |
|  | TLSTLAYMYDLLDPNVTF | Borrelia repeat protein motif | 1.1 | 595-612 | This presumed motif is functionally uncharacterized. | - |
|  | LNVPDLQQV | Coagulation Factor V LSPD Repeat motif | 8.3 | 366-374 | This presumed motif is functionally uncharacterized. | - |
| **Movement Protein** | KGFLSRQADKVKKKIYRCVGRVFLVYVPIIQATTSGLITLKLQNSDTGEISDVVTDVEV | 3A/RNA2 movement protein family | 0.33 | 69-127 | The 3A protein is found in movement proteins from Bromoviruses, Cucumoviruses and Dianthoviruses. The function of the 3A protein is uncertain but takes part in the cell-to-cell movement of the virus. | Canto et al., 1997 |
|  | KLQNSDTGEISDVVTDVEVNRAFVIMDRWGRSLVESADLNLLYSISCPDVRPG | Type 4 fimbrial biogenesis protein PilY2 | 0.27 | 109-161 | Members of this family were involved in fimbrial biogenesis, but its exact role is unknown. Fimbrial biogenesis genes of *Pseudomonas aeruginosa*- pilW and pilX increase the similarity of type 4 fimbriae to the GSP protein-secretion systems, and pilY1 encodes a gonococcal PilC homolog. | [R A Alm](https://pubmed.ncbi.nlm.nih.gov/?term=Alm+RA&cauthor_id=8899718) et al., 1996 |
|  | STSDFSVVECSMDEMSQISEDLHKLMLSDEMKALPTKGCHILHLVNLPKSNILRLASKEQKGFLSRQADKVKKKIYRCVGRVFLVYVPIIQATTSGLITLKLQNSDTGEISDVVTDVEVNRAFVIMDRWGRSLVESADLNLLYSISCPDVRPGARVGEMMVFWDERMSRQQTYLEKGNPILFPIAETKPSKYLNDKKVLMSMVRSR | Bromovirus movement protein | 2e-25 | 9-214 | Members of this family can be seen in the bromoviridae family, where they form lengthy tubular structures on the infected protoplast's surface. These proteins aid the infection of the virus. This motif enhances the ability of the movement proteins from the brome and alfalfa mosaic viruses to create tubules. | [D T Kasteel](https://pubmed.ncbi.nlm.nih.gov/?term=Kasteel+DT&cauthor_id=9267012) et al., 1997; s |
| **Coat protein** | FDTFARTHRVVIEFKTEVPAGAKVLVRDLYVVVSDLPRVQIPTDVLLVDEDLLEI | WD-3/WD-repeat region | 0.17 | 172-225 | The WD-3/WD-repeat region is Fifty-five residues have three WD repeats and six cysteine residues as three cysteine bridges. Human Fancl protein, the probable E3 ubiquitin ligase subunit of the FA complex, contains these domains (Fanconi anaemia). A multiunit nuclear complex composed of eight Fanconi anaemia gene products is required for the mono-ubiquitination of the FANCD2 FA protein, which is a downstream FA protein. For the FA complex's other subunits to interact with one another, the WD repeats are necessary. | Kominami et al., 1998 |
|  | NNPNRNRNPNRVSSGVGPAIR | The Domain of the unknown function (DUF4167) | 0.086 | 37-57 | This presumed Domain is functionally uncharacterized. | - |
|  | GGCRSCKKCHPNDALIPLRAQQRAVNNPNRNRNPNRVSSGVGPAIRPQPVVKTTWTVRGPNVPPRIPKGYVAHNHREVTTTEAVKYLSIDFTTTLPQLMGQNLTLLTVIVRMNSMSSNGWIGMVEDYKVDQPDGPNALSRKGFLKDQPRGWQFEPPSDLDFDTFARTHRVVIEFKTEVPAGAKVLVRDLYVVVSDLPRVQIPTDVLLVDE | Ilarvirus coat protein domain | 1.6e-101- 6.3e-101 | 12-221 | The Ilarvirus coat protein domain is conserved in various coat proteins from the ilarviruses of the Bromoviridae family. The ilarvirus coat protein must start replicating the viral genome in host plants. Bromoviridae members have a positive stand ssRNA genome and no DNA replication step. | [Sánchez-Navarro](https://pubmed.ncbi.nlm.nih.gov/?term=S%C3%A1nchez-Navarro+JA&cauthor_id=9170502) and  [Pallás](https://pubmed.ncbi.nlm.nih.gov/?term=Pall%C3%A1s+V&cauthor_id=9170502) 1997 |
